# Supplementary material for: Down syndrome with Alzheimer's disease brains have increased iron and associated lipid peroxidation consistent with ferroptosis
Source: Alzheimers Dement. 2025 Jun 19;21(6):e70322. doi: 10.1002/alz.70322 (PMC12177674; doi:10.1002/alz.70322)
Supplement: Supplementary file 2 — Supporting information [file ALZ-21-e70322-s002.docx]

| **Diagnosis** | **Genotype** | **Braak** | **Age** | **Sex** | **PMI** | **ADRC** |
| --- | --- | --- | --- | --- | --- | --- |
| CTL | ApoE3,3 | 0 | 82 | Male | 9 | USC |
| CTL | ApoE3,3 | 0 | 93 | Male | 12 | USC |
| CTL | ApoE3,3 | 0 | 93 | Male | 3.75 | USC |
| CTL | ApoE3,3 | 0 | 76 | Male | 11.25 | USC |
| CTL | ApoE3,3 | 0 | 99 | Female | 9 | USC |
| CTL | ApoE3,3 | 0 | 91 | Female | 8.75 | USC |
| CTL | ApoE3,3 | 1 | 95 | Female | 3.25 | USC |
| CTL | ApoE3,3 | 0 | 85 | Female | 7 | USC |
| AD | ApoE3,3 | 4 | 87 | Male | 4.75 | USC |
| AD | ApoE3,3 | 5 | 88 | Male | 6.75 | USC |
| AD | ApoE3,3 | 3 | 97 | Male | 5.25 | USC |
| AD | ApoE3,3 | 5 | 76 | Male | 9.75 | USC |
| AD | ApoE3,3 | 5 | 81 | Female | 7.5 | USC |
| AD | ApoE3,3 | 6 | 66 | Female | 17 | USC |
| AD | ApoE3,3 | 5 | 89 | Female | 1.5 | USC |
| AD | ApoE3,3 | 4 | 94 | Female | 10.5 | USC |
| DSAD | ApoE3,3 | 6 | 54 | Male | 4.3 | UCI |
| DSAD | ApoE3,3 | 6 | 49 | Male | 4 | UCI |
| DSAD | ApoE3,3 | 6 | 55 | Male | 3.18 | UCI |
| DSAD | ApoE3,3 | 6 | 70 | Male | 3.87 | UCI |
| DSAD | ApoE3,3 | 6 | 57 | Female | 4.25 | UCI |
| DSAD | ApoE3,3 | 6 | 63 | Female | 3.58 | UCI |
| DSAD | ApoE3,3 | 6 | 62 | Female | 3.8 | UCI |
| DSAD | ApoE3,3 | 6 | 56 | Female | 2.92 | UCI |
| mDS | ApoE3,3 | 6 | 54 | Male | 4.5 | UCI |
| mDS | ApoE3,3 | 3 | 48 | Female | 18.35 | UCI |
| mDS | N/A | 1 | 55 | Male | N/A | UCI |
| pDS | ApoE2,3 | 3 | 72 | Male | 4.87 | UCI |

**Supplemental Table 1:** Patient information for the tissue used in this study.


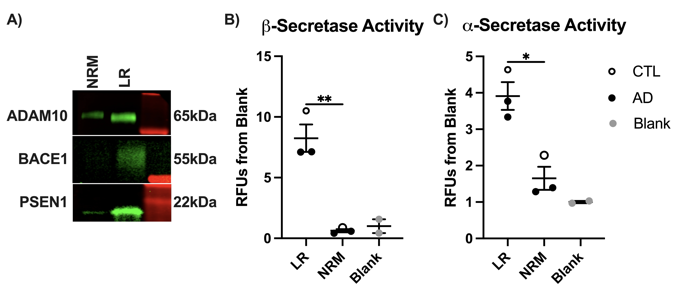


**Supplemental Figure 1**: Validations of Secretase localization and activities. **A)** comparison of NRM and LR fractions for ADAM10, BACE1, and PSEN1 by Western Blot in human prefrontal cortex. Comparison of NRM, LR, and blank wells (all reagents except sample) for **B)** β- secretase, and **C)** α-secretase activities presented as relative fluorescence from blank in human prefrontal cortex. t-test, *p<0.05, **p<0.01.


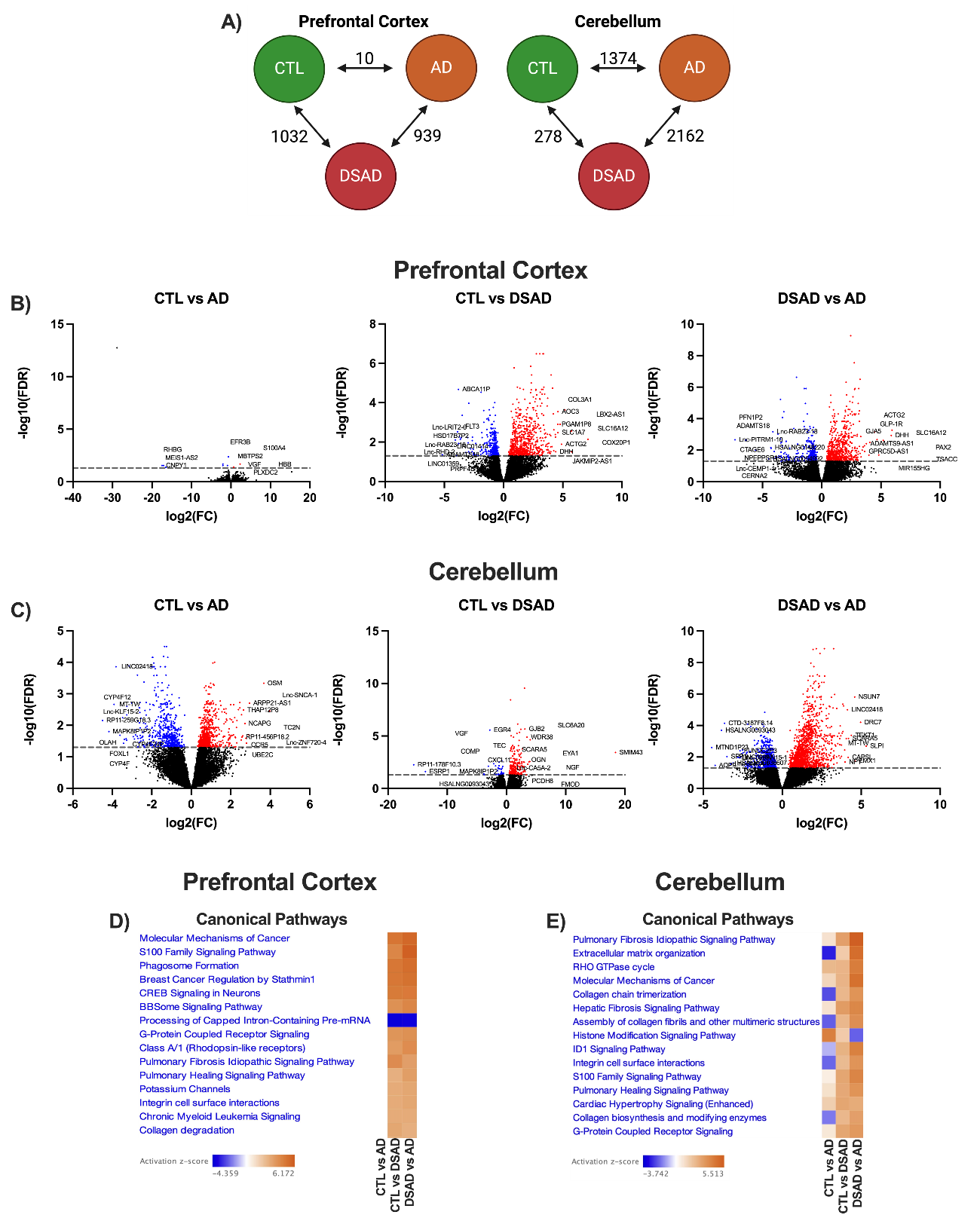


**Supplemental Figure 2:** Transcriptional changes with sporadic AD and DSAD. **A)** Shared differentially expressed genes (DEGs) between cognitively normal, AD, and DSAD for prefrontal cortex and cerebellum. Volcano plots showing the top 10 up and down-regulated genes for CTL vs AD, CTL vs DSAD, and DSAD vs AD for **B)** prefrontal cortex and **C)** cerebellum. The top 15 identified pathways altered in **D)** prefrontal cortex, and **E)** cerebellum.
